# ASSURE-CSU: a real-world study of burden of disease in patients with symptomatic chronic spontaneous urticaria

**DOI:** 10.1186/s13601-015-0072-9

**Published:** 2015-08-17

**Authors:** Karsten Weller, Marcus Maurer, Clive Grattan, Alla Nakonechna, Mohamed Abuzakouk, Frédéric Bérard, Gordon Sussman, Ana M. Giménez-Arnau, Javier Ortiz de Frutos, André Knulst, G. Walter Canonica, Kelly Hollis, Doreen McBride, Maria-Magdalena Balp

**Affiliations:** Allergie-Centrum-Charité, Department of Dermatology and Allergy, Charité – Universitätsmedizin Berlin, Charitéplatz 1, D-10117 Berlin, Germany; Dermatology Centre, Norfolk and Norwich University Hospital, Norwich, UK; Allergy and Immunology Clinic, Royal Liverpool and Broadgreen University Hospital, Liverpool, UK; Clinical Immunology and Allergology, CH Lyon-Sud, Claude Bernard University Lyon I - Faculty of Medicine, Lyon Sud, Lyon, France; Gordon Sussman Clinical Research Inc. 202 St Clair Avenue West, Toronto, Canada; Dermatology Department, Hospital del Mar, Institut Mar d’Investigacions Mèdiques, Universitat Autònoma, Barcelona, Spain; Hospital Universitario 12 de Octoubre, Dermatology Department, Madrid, Spain; Department of Dermatology and Allergy, UMC, Utrecht, The Netherlands; University of Genoa, San Martino-IST, Genoa, Italy; RTI Health Solutions, Raleigh, NC USA; RTI Health Solutions, Manchester, UK; Novartis Pharma AG, Basel, Switzerland

**Keywords:** Chronic spontaneous urticaria, Chronic idiopathic urticaria, Angioedema, Disease burden, Humanistic burden, Economic cost, Presenteeism, Absenteeism, Observational

## Abstract

**Background:**

Chronic spontaneous urticaria (CSU) formerly known as chronic idiopathic urticaria (CIU) is a severe and distressing skin condition that remains uncontrolled in approximately one half of patients, despite the use of licensed, recommended doses of modern, second-generation H_1_-antihistamines. So far, the humanistic, societal and economic burden of CSU/CIU has not been well quantified. Therefore it is important to broaden our understanding of how CSU/CIU impacts patients, society, and healthcare systems, by determining the disease burden of CSU/CIU and the associated unmet need; as well as to further guide the use of new treatments in an efficient and cost-effective manner.

**Methods:**

ASSURE-CSU is an observational, multicenter study being conducted in the UK, Germany, Canada, France, Italy, Spain, and The Netherlands. The study comprises a retrospective medical chart review in conjunction with patient surveys (including validated tools for assessment of disease impact) and an 8-day patient diary. The primary objectives of the study are to describe patient demographics, medical history, treatments, and healthcare resource utilization based on medical-record data and to assess the impact of disease, healthcare resource utilization, work days missed, and productivity loss based on patient-reported data. Approximately 700 patients (aged ≥18 years) will be enrolled who have CSU/CIU despite currently receiving treatment, and have had persistent symptoms for at least 12 months. Data will be collected retrospectively for the 12 months (±1 month) prior to enrolment wherever possible, and prospectively for the week following enrolment.

**Discussion:**

ASSURE-CSU will be the first study to examine the economic and humanistic burden of disease in patients diagnosed with CSU/CIU who are symptomatic despite treatment. By combining retrospective evaluation of medical records with prospective patient surveys and 8-day diaries, across seven different countries, the ASSURE-CSU study will contribute to a better understanding and acknowledgement of the burden of disease in patients with symptomatic chronic spontaneous urticaria.

## Background

Urticaria is characterized by the sudden appearance of hives, angioedema or both [1]. Chronic spontaneous urticaria (CSU), formerly known as chronic idiopathic urticaria (CIU) is defined by the spontaneous development of itchy hives and/or angioedema that reoccur for at least 6 weeks due to known or unknown causes [1]. CSU/CIU is thought to affect 0.5–1 % of the global population at any given time, with CSU/CIU accounting for approximately two-thirds of all cases of chronic urticaria (CU) [2]. It has been estimated that 33–67 % of patients with CSU/CIU have both hives and angioedema, with 29–65 % having hives alone and 1–13 % angioedema alone [2]. Although the clinical features and pathogenesis of CSU/CIU have been well studied over the past decade [3], the humanistic and economic burden of CSU/CIU have not been elucidated in detail, specifically, in patients with an inadequate response to first-line therapies.

However, generally across all patient types, CSU/CIU can have a considerable burden on patients, healthcare systems and society. Moreover, the current standard of care, H_1_-antihistamines at licensed doses, is only effective at resolving symptoms in less than 50 % of patients with CSU/CIU. Further increases in dose of H_1_-antihistamines does improve treatment response, but every third or fourth patient is thought to remain refractory (symptomatic despite treatment) [2], and may therefore have a higher burden of disease when compared to non-refractory patients. The burden of disease is also particularly high in patients with CSU/CIU and associated angioedema, which, when compared to hives alone, can increase disease severity and duration [2]. In addition to the impact of the physical signs and symptoms, CSU/CIU can also have a profound impact on the lives of patients due to factors such as sleep disruption, anxiety, embarrassment, depression and social isolation [2, 4–7]. Many aspects of daily activities (e.g., choice of clothes or going to the shops [8]) are also negatively impacted in patients with CSU/CIU [2]; and for those patients who are employed, impaired work performance or even absence from work are common [2, 9]. There is also a need to ensure timely and appropriate referral of patients with CSU/CIU to specialists in order to reduce delays in their diagnosis and the implementation of effective pharmacological management. This is especially true for those patients who are symptomatic despite on-going treatment with current standard of care. In many cases this need is not met, with patients seeing an average of more than two physicians before being referred to a specialist [10]. Reducing the burden associated with CSU/CIU therefore requires a broader understanding of the impact of these unmet needs of patients [2].

The guidelines recommend a stepwise approach in the management of CSU, beginning with licensed doses of H_1_-antihistamines [1]. However, many patients remain uncontrolled and in these situations, current guidelines recommend increasing the dose by up to four-fold [1]. In patients non-responsive to higher doses of H_1_-antihistamines, guidelines recommend the addition of a third-line treatment option of omalizumab, ciclosporin or montelukast [1]. Among these third-line options, only omalizumab is currently approved as add-on therapy for the treatment of CSU/CIU in adult and adolescent (≥12 years) patients showing an inadequate response to H_1_-antihistamine treatment.

Few studies have specifically evaluated the economic impact of CSU/CIU. In 2005, DeLong and colleagues estimated the total annual cost in the US as being $2047 per patient, with indirect costs accounting for 15.7 % ($322) [9]. Another study analyzing 2002 data estimated the total annual cost in France as being €2128 per patient, with productivity costs accounting for 92.2 % (€1962) [11]. Studies based on insurance claims cannot assess clinical aspects and patient perspectives because they lack recorded clinical data; it is also possible that the lack of specific International Classification of Diseases (ICD) code for CSU/CIU [12], may have resulted in an underestimated number of affected patients. As such, the true economic cost of CSU/CIU needs to include both direct costs, such as medication and costs of treatment, as well as indirect costs related to lack of productivity and absence from work.

Importantly, there are no studies which specifically evaluate the burden of disease in patients who are non-responsive to standard-of care (H_1_-antihistamines at licensed doses). The objective of ASSURE-CSU is to identify and quantify the humanistic, societal and economic burden of disease in patients with inadequately controlled CSU/CIU, with a focus on patients whose disease persists for ≥12 months. This study will evaluate health-related quality-of-life (HRQoL) using patient-reported outcomes (PROs), healthcare resource utilization, absence from work and productivity loss among patients with CSU who are currently symptomatic despite treatment. This study, collecting real-world data in this patient population, will contribute to a better understanding of the impact of CSU/CIU and its associated unmet need.

## Methods/design

### Study design and participants

**Fig. 1:**
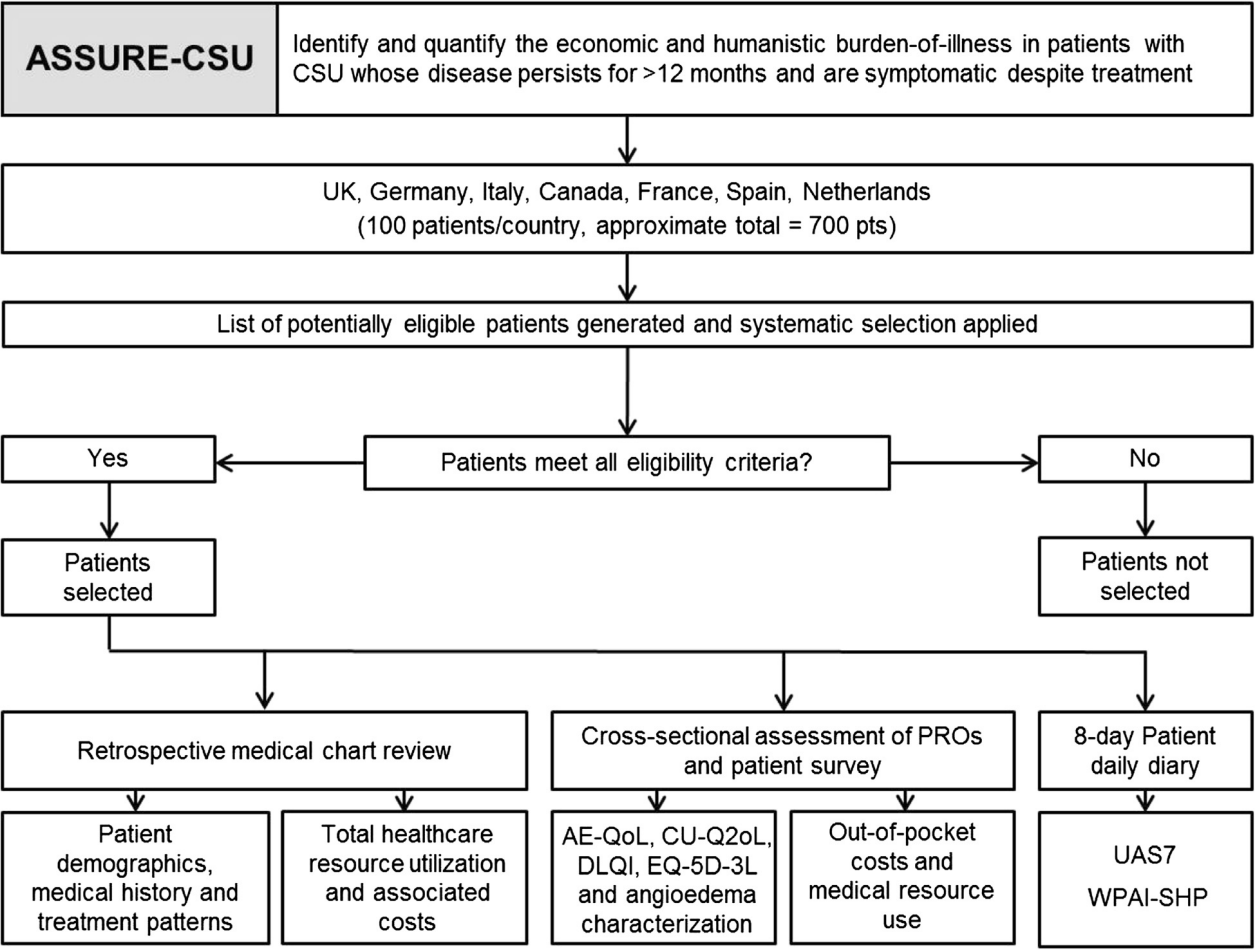
Study protocol. *CSU* chronic spontaneous urticaria; *PRO* patient-reported outcome; *AE-QoL* Angioedema Quality-of-Life Questionnaire; *CU-Q2oL* Chronic Urticaria Quality-of-Life Questionnaire; *DLQI* Dermatology Life Quality Index; *EQ-5D-3L* three-level EuroQoL-5D; *UAS7* Weekly Urticaria Activity Score; *WPAI-SHP* Work Productivity and Activity Impairment – Specific Health Problems

ASSURE-CSU is an observational, multinational, multicenter study being conducted in seven countries (UK, Germany, Canada, France, Italy, Spain and The Netherlands). The study has two components: a retrospective medical chart review including patient medical-record abstraction and a patient survey that contains several validated PROs (Fig. 1). These measures will assess disease and dermatological quality of life (QoL), general health status, symptoms and also the impact of CSU/CIU on work and activities. Planned enrolment is approximately 700 CSU/CIU patients (approximately 100 patients from an average of 10 centers in each participating country) who are at least 18 years of age, are symptomatic despite current treatment, and have symptoms that have persisted for at least 12 months. Inclusion and exclusion criteria are detailed in Table 1.

**Table 1:** Patient selection criteria in the ASSURE-CSU study

Inclusion criteria	Exclusion criteria
• Clinician-confirmed diagnosis of CSU/CIU, defined as occurrence of spontaneous hives and/or angioedema for at least 6 weeks without an obvious cause	• Symptoms resolved within 12 months of diagnosis
• Symptomatic for more than 12 months at least 3 days per week and currently symptomatic despite treatment	• Determined to be in remission/resolved, defined as more than 6 months untreated and without symptoms
• Under care of the participating site/physician for at least 12 months or the participating site/physician has documentation of the patient’s relevant medical information (e.g., referral letters) for the 12 months prior to enrolment	• Urticaria is predominantly of the inducible form (i.e., physical, cholinergic); however, patients with comorbid inducible urticaria may be included in the study provided that it is of secondary importance to CSU/CIU
• Received at least one treatment course with an H_1_-antihistamine (licensed dose)	• Participated in a CSU/CIU interventional study or clinical trial during the data collection period
• Able to provide informed consent	• Unable to provide informed consent
• Willing and able to complete the surveys	• Not able and not willing to complete the surveys
• Aged 18 years or older at the time of study enrolment	

*CSU* chronic spontaneous urticaria

### Objectives

The primary objectives of the study are to describe patient demographic characteristics, medical history, treatments and healthcare resource utilization based on medical record data; as well as impact of disease, healthcare resource utilization, work days missed and productivity loss based on patient-reported data (Table 2). Secondary objectives are to collect the following information from medical records and patient-reported data: number of patients receiving guideline recommended treatments (EAACI/GA^2^LEN/EDF/WAO [1]); diagnostic code or terminology used to identify CSU/CIU, as recorded in the medical records (e.g., chronic idiopathic/chronic ordinary versus chronic spontaneous urticaria); treatment response as available in the medical record; disease severity and symptoms linked to medical resources; loss of productivity and impact on patients’ HRQoL.

**Table 2:** Primary assessments of the ASSURE-CSU study

Medical record data	Patient-reported data
Demographic characteristics	Impact of disease tools:
	• EQ-5D-3L
	• DLQI
	• CU-Q2oL
	• AE-QoL (in patients with angioedema)
Baseline medical history	Angioedema history and characterization
Treatment received for CSU/CIU	Healthcare resource utilization (other than that recorded in charts, use of alternative medications and out-of-pocket expenses)
Duration of treatments	Disease activity (UAS7)
Total healthcare resource utilization	Work days and productivity loss (WPAI-SHP)

*EQ-5D-3L* three-level EuroQoL-5D, *DLQI* Dermatology Life Quality Index, *CU-Q2oL* Chronic Urticaria Quality-of-Life Questionnaire, *AE-QoL* Angioedema Quality-of-Life Questionnaire, *CSU* chronic spontaneous urticaria, *UAS7* Weekly Urticaria Activity Score, *WPAI-SHP* Work Productivity and Activity Impairment – Specific Health Problems

### Data collection

Clinical staff in tertiary care review patient medical records and capture demographic, clinical, and healthcare resource data, to be extracted via a web-based electronic data capture application. Retrospective data are collected for the previous 12 months (±1 month) prior to study enrolment. The following data are collected: patient demographics including age, gender, and ethnicity; baseline medical history including co-morbidities, duration and severity of CSU/CIU; treatments received for CSU/CIU including duration and treatment response; healthcare resource utilization related to CSU/CIU, including the type and specialty of healthcare professional visited and frequency of visits and the number of emergency room visits. Further details of the estimation of direct and indirect costs are summarized in Table 3.

**Table 3:** Evaluation of direct and indirect costs associated with CSU

Evaluation of costs
Data sources
• Medical record abstraction form
• Patient survey
Unit costs
Country-specific unit cost estimates will be applied where available, using standard national sources, e.g.,
• UK National Health Service Reference Costs
• German *Einheitlicher Bewertungsmaßstab* (Uniform Rating Scale)
• National drug formularies
• National average wage statistics
Direct health care costs (all CSU-related)
• Physician visits, including private practice visits
• Emergency room visits
• Hospitalizations
• Therapies (including drugs, alternative medicine and out-of-pocket expenses)
• Laboratory tests
• Transportation costs
Indirect costs (employed patients; CSU-related)
• Lost productivity due to sick leave and early retirement (analyzed using the friction cost approach)
• Reduced productivity due to reduced performance at work (presenteeism)

*CSU* chronic spontaneous urticaria

Patient-completed surveys and 8-day diaries collect information from all eligible patients. The cross-sectional surveys capture information on healthcare resource utilization related to CSU/CIU and specifically angioedema, if applicable. This includes the number of patient-reported medical visits (not recorded in medical record charts), as well as out-of-pocket costs including transportation/parking costs. Impact of disease will be assessed using four validated PROs: the Dermatology Life Quality Index (DLQI), Chronic Urticaria Quality-of-Life Questionnaire (CU-Q2oL), three-level EuroQoL-5D (EQ-5D-3L) and, in patients with angioedema, the Angioedema Quality-of-Life Questionnaire (AE-QoL). The DLQI is designed for use in patients with dermatological disorders and is not specific to CSU/CIU, but has been used previously across a number of dermatologic diseases (including CSU/CIU) and measures overall impact on HRQoL in such dermatological conditions [13, 14]. It has a seven day recall period and scores from 0 to 30 with higher score meaning higher impact and lower QoL.

The CU-Q2oL, which measures various aspects of chronic urticaria specific QoL impairment [15], has a two week recall period and a score from 0 to 100, with higher score indicating higher QoL impairment.

The EQ-5D-3L is a generic tool that is used to measure health status in a wide variety of diseases [16, 17]. It assesses the impact of disease in that day and allows the calculation of a utility score ranging from 0 (death) to 1 (perfect health).

The AE-QoL assesses the impact of angioedema on patient QoL [18]. In addition the angioedema survey allows a description of angioedema episodes with their frequency, localization, symptoms and duration.

The patient diary collects seven days of information on disease activity using the Urticaria Activity Score (UAS) and on day 8, work absence and productivity information using the Work Productivity and Activity Impairment-Specific Health Problems (WPAI-SHP) questionnaire. The UAS is a validated diary-type tool that daily assesses disease activity through the severity of symptoms (hives and itch) [19]. It is usually scored over a period of seven days to give the weekly UAS score (UAS7) ranging from 0 to 42, with higher scores indicating greater disease severity [20]. The validity and reproducibility of the WPAI-SHP questionnaires are well established in a number of specific diseases (including the dermatological condition chronic hand dermatitis) [21]. This questionnaire captures absence from work (absenteeism), reduced productivity while at work (presenteeism), overall activity impairment and has a recall period of seven days.

### Data analyses

All study results will be summarized descriptively for each country (apart from direct and indirect costs which will estimated for each country using validated local sources) and for the pooled population across all countries. This includes a tabular display of mean values, medians, ranges, standard deviations for continuous variables of interest and frequency distributions for categorical variables. Results will be stratified by disease severity as measured by the UAS7 score.

For data on healthcare resource utilization, costs will be calculated for items by estimated unit costs for each resource per country. All cost data will be inflated to the most current cost year (2013/4) and adjusted to the currency value for each country where appropriate. Indirect costs will be estimated using the WPAI results for employed patients only, which uses an algorithm relevant for each country, based on average number of weekly hours worked and the hourly country-specific wage. The total indirect cost, presented also as overall work impairment cost, incorporates both absenteeism and presenteeism related costs.

In addition to descriptive statistical summaries of study results, where appropriate, multivariate analyses may be conducted to assess the differences in resource utilization and cost outcomes between levels of disease severity.

## Discussion

Currently available literature points to a significant humanistic and economic burden for patients with CSU/CIU as well as considerable demands on healthcare resources [2, 5–7, 9]. As up to 50 % of patients do not fully respond to currently recommended first-line therapy (H_1_-antihistamines at licensed doses) [2], further analysis is required to fully understand the disease burden in this particular subgroup of patients. ASSURE-CSU is the first study to address this unmet need. Its design will allow collection of medical resource utilization data and patient perspectives on a disease with an unrecognized burden. A better understanding of patient journey, time between onset, diagnosis and management will hopefully result in an optimized approach to patient management.

ASSURE-CSU does have some limitations which can be listed. Firstly, the study is only for patients with CSU/CIU who are symptomatic despite treatment with current standard of care (H_1_-antihistamines at licensed doses). Results are obtained from large treatment centers within each participating country, as such, information on patients who are not currently being treated or that are being treated by an office-based dermatologist/allergist will not be captured. Furthermore, it should be noted that patients may be symptomatic due to additional factors such as poor treatment compliance, under-treatment or treatment which has not been optimized for the patient.

The quantity and quality of the data obtained by medical record abstraction will be dependent on availability and accuracy of the data in the physicians’ records (e.g., duration of treatment, type of therapies or the relative success of previous treatments). As only 12 months of retrospective data are collected, a full picture of the patient since diagnosis is not obtained. A record of patient symptoms is collected prospectively for seven days using the UAS but this will only provide a snapshot of disease severity related to the UAS7 score of the representative week; this is particularly important to note, as disease severity can vary, and symptoms resolve over time.

Finally, although the ASSURE-CSU study has a broad number of objectives, because of its cross-sectional approach, it will not provide data on patient follow-up, natural disease history, natural remission rates, prevalence of CU or CSU/CIU, proportion of refractory patients, or proportion of responders to each therapy (within a country or globally). Neither will information on those patients who have H_1_-antihistamine-responsive CSU be collected, as they are outside of the focus of this analysis.

As far as we are aware, ASSURE-CSU will be the first international multicenter study to examine the economic and humanistic burden of disease on patients who are symptomatic despite current recommended first-line therapy. By using a combination of retrospective (patient medical-record abstraction) and prospective (patient surveys and 7-day diary) analysis across seven different countries, the ASSURE-CSU study can increase the understanding of the impact of CSU/CIU on patients and the associated costs. ASSURE-CSU will inform us of the patient journey, the potential success of management for refractory CSU/CIU and also possible ways to improve the perspectives that different stakeholders have on this disease. The study has been completed in five countries and data collection in the remaining countries is planned to be completed by the end of April 2015.

## Abbreviations

ASSURE-CSU: Assessment of the economic and humanistic burden of chronic spontaneous/idiopathic urticaria patients
AE-QoL: Angioedema Quality-of-Life Questionnaire
CIU: Chronic idiopathic urticaria
CSU: Chronic spontaneous urticaria
CU-Q2oL: Chronic Urticaria Quality of Life Questionnaire
DLQI: Dermatology Life Quality Index
EQ-5D-3L: three-level EuroQoL-5D
PRO: Patient-reported outcomes
UAS7: Weekly Urticaria Activity Score
WPAI-SHP: Work Productivity and Activity Impairment – Specific Health Problems
